# Progesterone-responsive vaginal leiomyoma and hyperprogesteronemia due to ovarian luteoma in an older bitch

**DOI:** 10.1186/s12917-020-02507-z

**Published:** 2020-08-10

**Authors:** L. Ferré-Dolcet, S. Romagnoli, T. Banzato, L. Cavicchioli, R. Di Maggio, A. Cattai, M. Berlanda, M. Schrank, A. Mollo

**Affiliations:** Department of Animal Medicine, Production and Health, University of Padua, Padua, Italy

**Keywords:** Leiomyoma, Aglepristone, Prostaglandin, Luteoma

## Abstract

**Background:**

This is the first report about a vaginal leiomyoma concomitant with an ovarian luteoma in a bitch.

**Case presentation:**

A 11-year-old intact female Labrador retriever was referred because of anuria, constipation and protrusion of a vaginal mass through the vulvar commissure. The bitch had high serum progesterone concentration (4.94 ng/ml). Because of the possibility of progesterone responsiveness causing further increase of the vaginal mass and since the bitch was a poor surgical candidate a 10 mg/kg aglepristone treatment was started SC on referral day 1. A computerized tomography showed a 12.7 × 6.5 × 8.3 cm mass causing urethral and rectal compression, ureteral dilation and hydronephrosis. A vaginal leiomyoma was diagnosed on histology. As serum progesterone concentration kept increasing despite aglepristone treatment, a 0.02 ng/mL twice daily IM alfaprostol treatment was started on day 18. As neither treatment showed remission of clinical signs or luteolysis, ovariohysterectomy was performed on referral day 35. Multiple corpora lutea were found on both ovaries. On histology a luteoma was diagnosed on the left ovary. P4 levels were undetectable 7 days after surgery. Recovery was uneventful and 12 weeks after surgery tomography showed a reduction of 86.7% of the vaginal mass. The bitch has been in good health and able to urinate without any complication ever since.

**Conclusions:**

This case demonstrates the importance of identifying progesterone related conditions as well as the importance of judiciously using a combined medical and surgical approach.

## Background

Several reproductive conditions have been described to be induced in bitches by ovarian steroidal hormones such as mammary, vaginal and vulvar tumors, vaginal prolapse or the cystic endometrial hyperplasia-pyometra complex [1, 2]. Tumors affecting the canine vaginal vestibule or vulva are rare, accounting for 2.4–3% of all tumors occurring in the dog and being mostly benign mesenchymal tumors such as leiomyomas or fibromas [3–5]. These tumors are considered to be non-invasive, slow-growing, smooth-muscle derived masses which do not cause metastasis in other organs [6, 7]. Ovarian tumors are reported to be uncommon in dogs [8–11]. Sex cord-stromal tumors have the ability to be hormonally active [12].

Surgical excision of vaginal masses through episiotomy has been described as the treatment of choice [13–15]. However, because of a) the old age of these patients and the risk performing extensive episiotomy and b) the fact that the growth of these masses may be stimulated by ovarian steroids, the use of steroidal hormones receptor antagonists or surgical ovariectomy have been reported as successful treatments [16, 17]. Aglepristone (AGLE) (Alizin®, Virbac) is a synthetic antiprogestogen marketed as an abortifacient in bitches in Europe and Australia and its off-label use for progesterone-related canine conditions has been reported [18]. Alfaprostol is a synthetic prostaglandin F2apha (PGF2α) compound marketed for use in food animals and reported to be able to induce luteolysis also in bitches [19].

## Case presentation

**Fig. 1:**
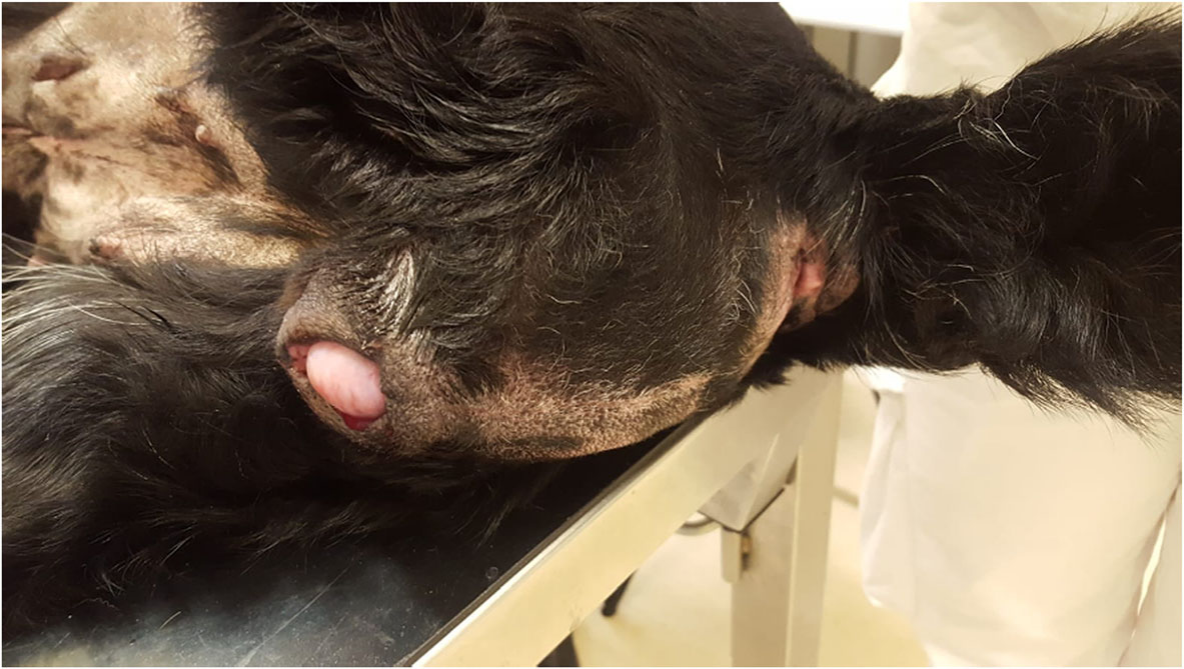
Large ovoid-shaped vaginal mass prolapsing from the vulvar commissure of an 11-year old intact Labrador bitch

**Fig. 2:**
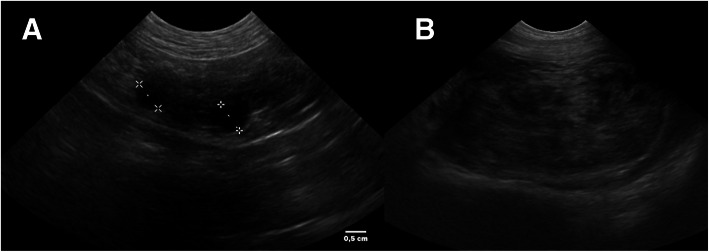
**a** Ultrasonography of the right ovary presenting preantral follicles of 0,7 cm. **b** Ultrasonography of the perineal area presenting the vaginal mass encapsulated in the cranial vagina

**Fig. 3:**
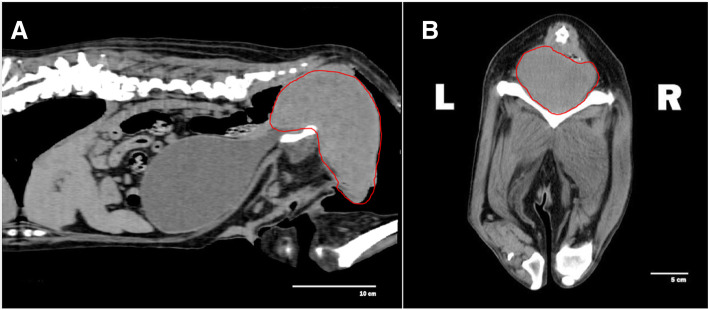
**a** Sagital image from a computerized tomography showing the vaginal mass measuring 12.72 cm in length, arousing from the cranial vagina and protruding from the pelvic canal. The urinary bladder shows to be enlarged due to the urethral compression. **b** Transversal image from a computerized tomography showing the presence of the vaginal mass of 6.52 cm in height and 8.33 cm in width, leaning on the pelvic bones provoking urethral compression

**Fig. 4:**
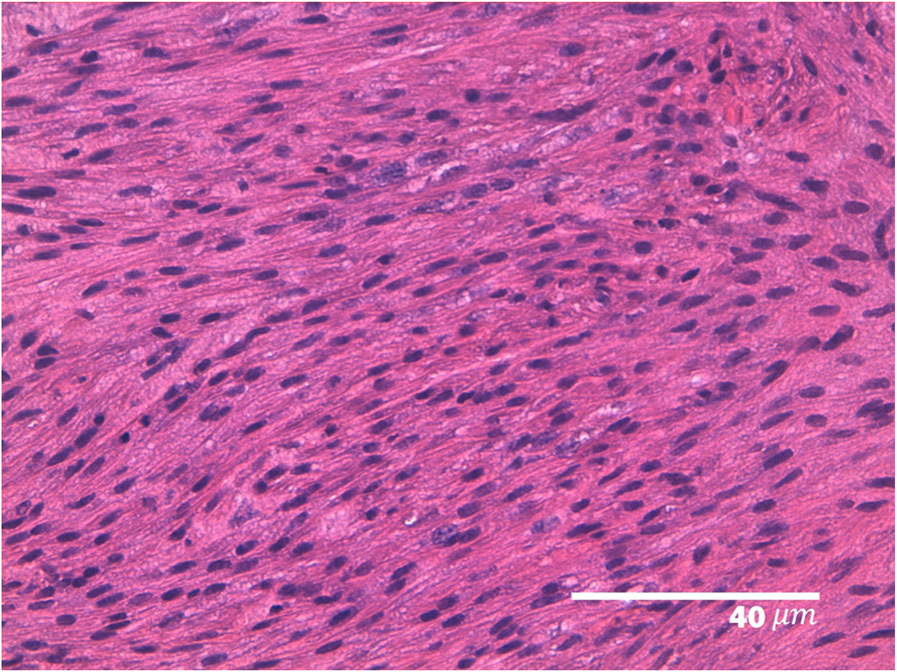
Histological image from the vaginal biopsy. The image presents a thin outer layer of mesenchymal fibrous tissue with a cellular population represented by spindle elements with defined margins and an eosinophilic cytoplasm. The image is compatible with a leyomioma

A 11-year old intact Labrador Retriever bitch was referred to the Veterinary Teaching Hospital of the University of Padova (Italy) because of a vaginal mass prolapsing from the vulva (Fig. 1). The bitch had a history of regular cycling every 8 months, had never been pregnant, her vaccination and heartworm prevention programs were current and had had no other health problem until shortly after the onset of her most recent proestrus (1 week prior to referral) when she started to become anuric and constipated. Because of constipation, her diet was changed to a commercial fiber diet. Presence of perineal swelling, an abundant bloody and muco-purulent vaginal discharge and a mass protruding from the vulva were first noticed 3 days after onset of proestrus. On clinical exam on the day of referral (day 1) the bitch was alert and normally responsive and her clinical parameters (pulse, respiration and rectal temperature, skin and subcutis and palpable lymph nodes) were normal. Her perineal area appeared enlarged and a rounded mass was protruding from the vulvar labia, covered by pink translucent vaginal mucosa. On digital palpation the mass had a very hard consistency and it was impossible to insert the operator’s finger beyond the vestibulum due to the mass occupying the entire vestibular lumen. Vaginal cytology was performed and stained with Diff Quick (RAL diagnostics, Milan, Italy) showing a 1:1 rate of intermediate and keratinized cells with abundant neutrophils and a small percentage of red blood cells. Abdominal ultrasonography showed a normal appearance of the uterine wall and mucosa and lack of fluid in the uterine lumen; several antral follicles of < 0.7 cm diameter were detected in both ovaries (Fig. 2a). Ultrasonography of the perineal area showed the mass to be originating from the cranial vagina and invading the vestibulum (Fig. 2b). Complete blood count revealed mild leukocytosis (22.10 × 10^3^/μl); biochemistry was unremarkable. Serum progesterone (P4) concentration measured with immunofluorometry (AIA 369 TOSOH, FUTURLAB s.r.l., Padova, Italia) was 4.94 ng/mL demonstrating the presence of luteal tissue. Because of the progressive increase in mass size after the onset of proestrus and considering the potential presence of P4 receptors within the mass [17] a 10 mg/kg dose of the P4 receptor antagonist AGLE was administered subcutaneously on day 1 and repeated the following day. Such treatment has been shown to reduce the size of a vaginal leyomioma by 50% in an older crossbreed bitch allowing for an easier surgical removal [17]. Antibiotic therapy with enrofloxacin (5 mg/kg) for 7 days was prescribed. On day 8 after presentation vaginal edema was slightly decreased in volume and the mass was not protruding from the vulva; however, it was still causing swelling of the perineum and on vaginal palpation its consistency was unchanged. The owner reported that constipation had improved starting from 48 h after the second AGLE injection, but the bitch was still anuric and with an abundant purulent vulvar discharge. On hematology and biochemistry the only abnormality was leukocytosis (37.98 × 10^3^/μl) while all other parameters (including kidney function) were normal. P4 concentrations had increased to 22.4 ng/mL. On abdominal ultrasonography the reproductive system showed no change while dilatation of the urinary bladder and ureters was observed which was causing hydronephrosis. A Foley catheter was inserted into the urethra and the bladder was emptied. A 10 mg/kg AGLE dose was administered and a combination of enrofloxacin and amoxicillin-clavulanic acid (5 mg and 15 mg/kg, respectively) twice daily was prescribed for another week to replace enrofloxacin as empiric treatment until urinary results would be available. On day 11 a total body computed tomography (CT) was performed under intravenous anaesthesia in order to evaluate size and position of the mass and determine whether other internal organs were affected. After premedication with 0.03 mg/kg acepromazine (Fatro, Italy) and 0.1 mg/kg methadone (Dechra, The Netherlands) intramuscularly, propofol 1% (Merial, Italy) was administered using a target controlled infusion system to induce and maintain anesthesia [20]. A large, ovoid shaped, heterogeneous, moderately contrast enhancing (pre- and post-contrast enhancement 32 ± 9 HU and 63 ± 9 HU, respectively) mass, measuring 12.72 cm in length, 6.52 cm in height and 8.33 cm in width, was visible on CT. The mass aroused from the vaginal wall from the middle third of the vagina and vastly protruded out from the pelvic canal and vagina. The rectum was displaced dorsally and the pelvic urethra ventrally. The urinary bladder was markedly distended reaching the cranial margin of L3. Both ureters were dilated (0.72 cm) and bilateral pyelectasis was evident (Fig. 3a-b). Regional lymph nodes were unremarkable. In addition to CT, an ultrasound guided transcutaneous needle biopsy was performed using a 20 G X 100 mm (ZAMAR s.r.l., Mantova, Italy). Pressure controlled mechanical ventilation and main cardiovascular and respiratory variables monitoring were guaranteed throughout both procedures. Histopathology revealed a thin outer layer of mesenchymal fibrous tissue with a cellular population represented by spindle elements, lacking defined margins with an eosinophilic cytoplasm. Histological patterns were compatible with a diagnosis of leyomioma (Fig. 4). The bitch was sent home with a urinary Foley catheter positioned in situ. On day 15 ureters and kidneys showed normal ultrasonographic patterns. Antibiotic theraphy was stopped at this time. The mass had apparently stopped growing. As P4 concentration had reached 39.5 ng/mL another 10 mg/kg dose of AGLE was administered. On day 18, P4 levels measured with immunofluorometry were > 40 ng/mL. As 40 ng/mL is the upper range of the immunofluorometry system, a dilution of 1:4 was performed in order to determine real P4 concentration which was 124 ng/mL. Due to such increased P4 secretion, an IM treatment course with 0.02 ng/kg of the synthetic PGF2α alfaprostol (Gabbrostim®, CEVA-VETEM, Milan, Italy) was started twice daily. The Foley catheter was removed. On day 20, hydronephrosis was again observed on ultrasonography. Serum P4 was 39 ng/mL (following samples dilution 1:1). Owners were instructed on how to perform manual abdominal compression to empty the bladder at home. On day 22 urine from the bladder was collected by cystocentesis and a urinalysis and urine culture with antibiogram were performed revealing a specific weight of 1.008 and a high population of multi-resistant Klebsiella spp. sensible only to trimethoprim-sulfonamide (TMP/SMX). The bitch received another 10 mg/kg dose AGLE and was started on an oral twice daily treatment with TMP/SMX (4 mg/kg and 20 mg/kg, respectively).

**Fig. 5:**
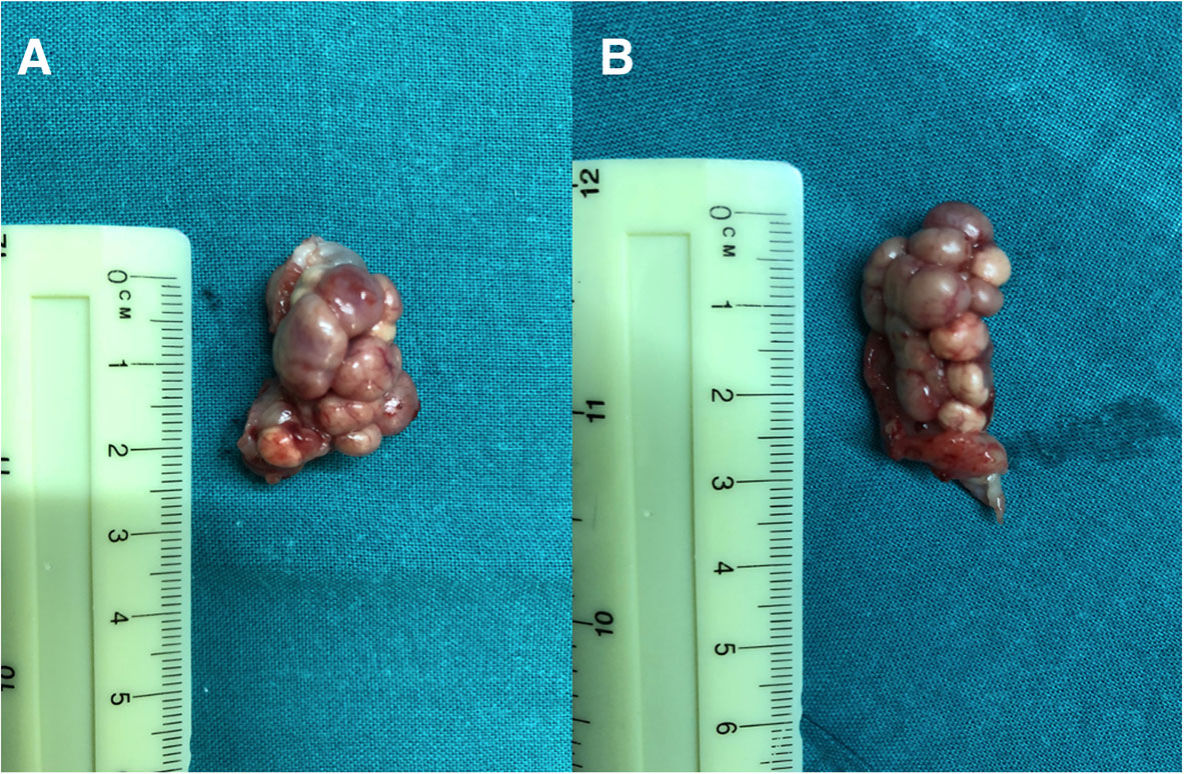
**a** Macroscopical image of the left ovary presenting 11 corpora lutea. **b** Macroscopic image of the right ovary presenting 12 corpora lutea

**Fig. 6:**
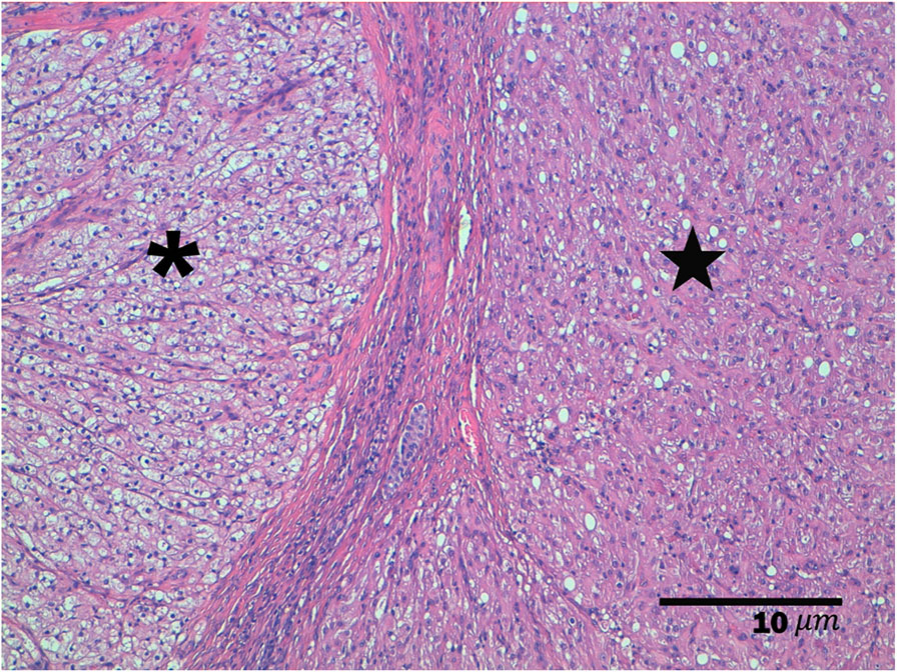
Histological image of the left ovary presenting a normal corpora lutea (astherisc) and a non-encapsulated and non-infiltrative nodular lesion composed of elements arranged in rugs and separated by thin fibrovascular septa (star). The cells of the nodular lesion are polygonal and occasionally as palisade with moderate and abundant cytoplasm variably vacuolized with round and central nucleus. The cells present a light degree of atypia without mitosis. A diagnosis of luteoma is compatible

**Fig. 7:**
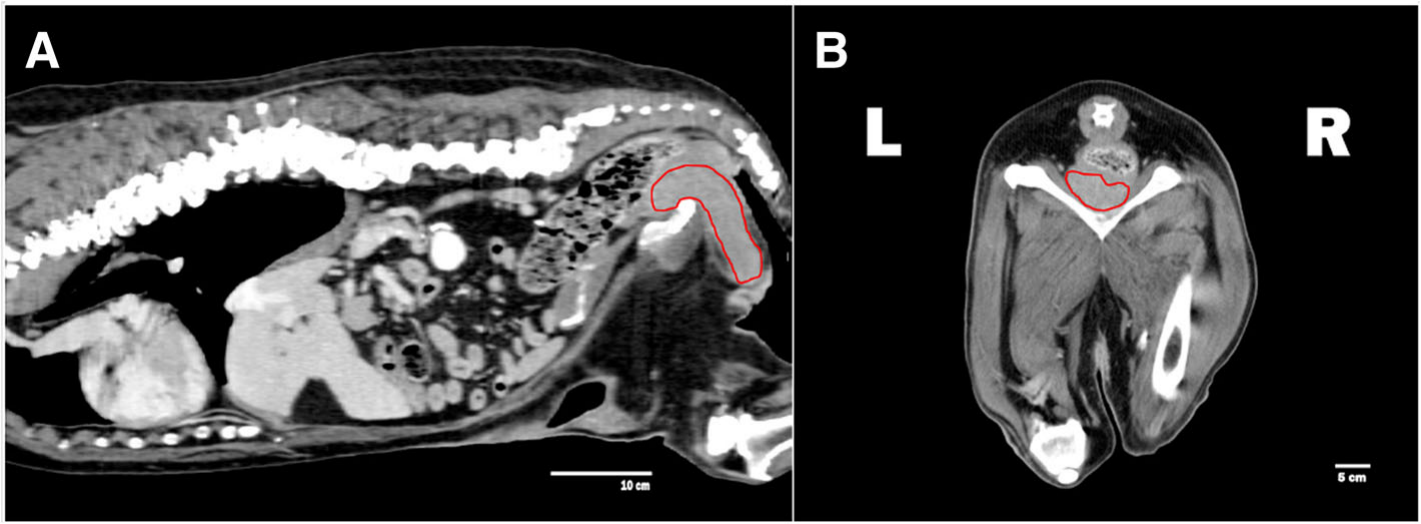
**a** Sagital image from a computerized tomography showing the reduction of the vaginal mass (9463 cm length) on the pelvic canal. The urinary bladder shows to be empty. **b** Transversal image from a computerized tomography showing the presence of the reduced vaginal mass of 2,7 cm height and 3,6 cm width. Dorsally to the mass rectum can be observed

On day 29 the bitch was clinically normal but the mass was still present without any relevant change in size. Serum P4 was 61 ng/mL (following 1:1 dilution) in spite of a 10-day twice daily treatment course with PGF2α. Another 10 mg/kg dose AGLE was administered. Because of the failure of luteolysis and fearing a P4-led progressive increase of the vaginal mass causing irreversible renal damage, ovariohysterectomy (OHE) was planned. On day 36 the bitch was sedated and anesthetized using the same protocol adopted for the CT scan on day 11. OHE was uneventful and the bitch recovered well. On macroscopical assessment a total of 23 corpora lutea were counted, 11 on the left ovary and 12 on the right ovary (Fig. 5a-b); the uterine body and uterine horns did not reveal any alterations. Following fixation in 4% paraformaldehyde, histology of the uterus revealed a superficial multifocal endometrial hemorrhage with no other alterations; the right ovary showed the presence of multiple normal corpora lutea; the left ovary showed multiple corpora lutea and a delimitated, non-capsular and non-infiltrative nodular lesion of 0,3 cm diameter composed of elements arranged in rugs and separated by thin fibrovascular septa. The cells appeared polygonal and occasionally palisade with moderate and abundant cytoplasm variably vacuolized with round and central nucleus; a light degree of atypia without mitosis was present. Such histological patterns were compatible with a diagnosis of luteoma (Fig. 6). Once diagnosis of the ovarian tumor was done, serum samples were used for testosterone detection (due to the fact that human luteomas are androgen secretory in 30% of the cases [21]) revealing testosterone levels of < 0.2 ng/mL in every sample performed. On day 45 the bitch was clinically normal, very active and was now able to urinate spontaneously. Serum P4 levels were not detectable and the mass showed to be significantly reduced but still palpable in vagina. No bacterial growth could be observed on a cystocentesis urine sample. On day 56 the bitch returned for a recheck. On physical exam her clinical parameters were normal but developed skin lesions on her chin over the last 3–4 days showing a deep pyoderma with furunculosis. Aerobic and anaerobic bacteriology was performed from vagina, urine and skin of the chin. Urine culture and swabs from vagina were negative while skin bacteriology revealed the presence of a coagulase-positive Staphylococcus spp. and a Klebsiella spp. both sensible to amoxicilin-clavulanic acid. The dog was treated with a combination of amoxicillin-clavulanic acid at the dose of 20 mg/Kg twice daily for 14 days. On day 125 (3 months post-OHE) another CT was performed to assess the vaginal mass whose volume was markedly reduced, measuring 9.5 cm in length, 2.7 cm in height and 3.6 cm in width. The urinary bladder was empty and the pelvic urethra and both ureters were within normal limits. No pyelectasis was evident (Fig. 7a-b).

## Discussion and conclusion

History, clinical findings, endocrine tests and vaginal histology of this case are all consistent with a diagnosis of vaginal leiomyoma sensitive to steroid hormones. Because of the old age of the dog and the size and position of her vaginal mass requiring extensive episiotomy, surgical removal of the mass was not our first choice. AGLE has been used successfully to reduce the size of a vaginal fibroma in an adult bitch [17]. Therefore, a total of six 10 mg/kg AGLE injections were administered to this bitch. Serum P4 continued to increase during AGLE treatment. Such an increase was unexpected. AGLE is known to leave serum P4 concentrations unaltered [22] unless treatment occurs during mid-diestrus at which time luteolysis is reported to occur [23]; This bitch was at the beginning of her diestrus when she received her first AGLE administration, therefore we did not expect luteolysis to occur immediately; instead she showed a marked serum P4 increase following treatment with AGLE, something which has never been reported. We feared that the 10 mg/kg AGLE dose might be insufficient to deal with the unusually high serum P4 concentrations of this bitch. Normal serum P4 concentration during the canine diestrus have been reported to range between 10 and 30 ng/mL [24–26]. The decision to start a medical treatment with the synthetic PGF2α alfaprostol and subsequently to opt for OHE was driven by a) considerations on the importance of blocking the action of endogenous P4 to help management of these conditions [17], b) concern about the unusually high serum P4 concentrations despite treatment, c) fear that increasing serum P4 concentrations might cause worsening of urinary conditions and d) no signs of luteolysis were observed at ovarian level during ultrasound controls. The fact that 12 weeks after OHE the mass was reduced by 86.7% is an indirect confirmation that progesterone played a role in the development of this condition. The ovarian luteoma present on the left ovary was likely responsible for the failure of AGLE+ PGF2α treatment to cause luteolysis, probably due to the unusual high concentrations of serum P4 secreted by the ovary. Ovariohysterectomy is known to reduce the size of hormone-sensitive vaginal masses [16, 17]. Surgery is certainly the treatment of choice in these cases, however the surgical approach was not considered initially because of the old age of this bitch and the owner’s concern about its related risks. In addition, the reduced vaginal mass was left in situ because its presence did not interfere to the bitch to continue with a healthy life.

Because of the amount of bladder distension, a complicated cystitis with bladder wall invasion was suspected. Therefore, the initial choice of an antibiotic treatment for this bitch was enrofloxacin due to the ease and speed with which this drug achieves tissue penetration [27, 28]. When urinalysis was performed at day 8 of referral, the bitch was hyposthenuric (urinary specific weight of 1008) and no bacteria were present in the sediment. However, it has been described that low urinary specific weights might create a false negative bacteriuria in the sediment [29]. The reason for adding amoxicillin-clavulanic acid a week later was to broaden the spectrum of coverage because of the increasing leukocytosis and the suspicious false negative bacterial cystitis. The use of fluoroquinolones and amoxicillin-acid clavulanic should be guided by culture and sensitivity results. Unfortunately, this was not done. A multi-resistant Klebsiella was found growing in urine culture on day 22: such a bacteria was certainly present in the bladder of this bitch already upon presentation although it is unknown whether or not it was already multi-resistant or if its multi-resistant status developed following the combined use of enrofloxacin and amoxicillin-clavulanic acid. As *E. coli* is frequently identified as a cause of cystitis and because of the close proximity of the prolapsed vaginal tissue with the anus, the occurrence of a complicated multibacterial ascending cystitis with *E. coli* and Klebsiella spp. upon presentation cannot be ruled out as the combined enrofloxacin and amoxicillin-clavulanic acid antibiotherapy may have eliminated the E.coli population.

The size of the vaginal mass of this bitch did not decrease during treatment unlike what happened in the case of Rollon et al.*,* (2007) who observed a 30% reduction of a 9 × 5 cm vaginal mass after 4 weeks of AGLE treatment. The larger size of the vaginal mass of this case (12.7 × 6.5 × 8.3 cm) plus the high serum P4 concentration may account for failure to cause reduction in mass size despite treatment. Following surgery, the vaginal mass decreased fairly rapidly with the bitch being capable of urinating spontaneously already 7 days following surgery. Such a rapid improvement (faster than what reported by Rollon et al.*,* 2007) underlines the difference in the clinical effect on the target organ when comparing the use of P4 receptor blockers to the surgical removal of P4 production.

Luteomas are considered sex cord-stromal tumors potentially able to secrete steroid hormones [3, 4, 12]. Sex cord-stromal tumors seem to be the most commonly reported ovarian tumors in the female with the least and the most common species being sows and queens, respectively [30]. These tumors seem to be derived from the luteal cells of ovarian stroma [4, 9]. Human luteomas are rare benign tumors occurring either in pregnancy or after menopause [31]. Human gestational luteomas are spontaneously regressing tumors which may cause virilization of female fetuses due to their androgen secretion [21, 32]. Only two cases of canine ovarian luteoma have been described [12, 33] with both cases developing from ovarian remnants in spayed bitches. The histological pattern of the ovary of this case (polyhedral cells with abundant vesicular cytoplasm) and the unusual secretion of P4 was compatible with an ovarian sex-cord tumor resembling luteoma.

To our knowledge this is the first report about a vaginal leiomyoma concomitant with an ovarian luteoma in a bitch with uncommon secretion of P4. This case demonstrates the importance of identifying and characterizing P4 related conditions as well as the importance of judiciously using a combined medical and surgical approach. OHE should always be considered when dealing with small animal reproductive conditions occurring in diestrus. This is particularly true when dealing with older patients in which an elective, uncomplicated OHE may be better than a longer and more invasive surgery. A medical approach using a P4-receptor blocker and/or a luteolytic agent may be indicated for patients who are poor surgical candidates.

## Data Availability

The datasets used and/or analyzed during the resolution of the case are available from the corresponding author on reasonable request.

## Abbreviations

AGLE: Aglepristone
PGF2α: Prostaglandin F2 alpha
P4: Progesterone
CT: Computed tomography
TMP/SMX: Trimethropim-sulfonamide
OHE: Ovariohysterectomy
